# Photocatalytic degradation data of benzene and toluene by ZnO coated on glass plates under simulated sunlight

**DOI:** 10.1016/j.dib.2018.08.040

**Published:** 2018-08-21

**Authors:** Ahmad Jonidi Jafari, Roshanak Rezaei Kalantari, Majid Kermani, Masoumeh Hasham Firooz

**Affiliations:** a Research Center for Environmental Health Technology, Iran University of Medical Sciences, Tehran, Iran; b Department of Environmental Health Engineering, School of Public Health, Iran University of Medical Sciences, Tehran, Iran

**Keywords:** Toluene, Benzene, Air pollution, Nanophotocatalytic oxidation, ZnO, Simulated sunlight

## Abstract

For this data article the photocatalytic oxidation of benzene and toluene by ZnO nanoparticles coated on glass plates were studied under simulated sunlight. ZnO nanoparticles were coated on three glass plates by heat attachment methods. To evaluate the photocatalytic removal of benzene and toluene, coated plates irradiated by metal halide lamp in a rectangular reactor in batch mode. The effect of initial pollutants concentration, temperature, relative humidity, irradiation time, concentration of zinc oxide suspension, were assessed. The surface morphology and structure of ZnO nanoparticles and ZnO coated on glass plates were characterized by scanning electron microscopy, X-ray diffraction and Field Emission Scanning Electron Microscopy. Sampling and analysis of pollutants were performed according to the National Institute for Occupational Safety and Health (NIOSH) method. To analyze the concentration of benzene and toluene, gas chromatography with flame ionization detector (GC-FID) was used. The data results indicated that photocatalytic process by ZnO under irradiation of metal halide lamp could remove benzene and toluene at optimum experimental conditions. Coating of glass surfaces by ZnO suspension, resulted in 46% and 57% removal of benzene and toluene as concentration of 50 ppm at 45 °C, and relative humidity of 40% after 240 min irradiation of metal halide lamp.

## Specifications Table

**Table:** 

Subject area	**Chemistry, Environmental Sciences and Engineering**
More specific subject area	Photocatalytic process
Type of data	Table, image, figure
How data was acquired	GC-FID, XRD, SEM, FE-SEM
Data format	analyzed
Experimental factors	photocatalytic experiment was assessed at 45 °C, and relative humidity of 40% after 240 min irradiation of metal halide lamp
Experimental features	ZnO powders were immobilized on glass plates by heat attachment method
	The photocatalytic oxidation of benzene and toluene were carried out in a rectangular reactor under metal halide lamp irradiation
	The pump׳s airflow was divided into two parts, the impinger containing the pollutant liquid to supply contaminated air and humidifier
	After the photocatalytic experiments, the inlet and outlet of the reactor were closed and the reactor was sampled
Data source location	Tehran, Iran, Iran University of Medical Sciences, air pollution lab
Data accessibility	Data are accessible with article

## Value of the data

• Method and data will be advantageous for scale up and design the photocatalytic experimental set up for removing VOCs.
• The data provide information about photocatalytic oxidation of VOCs by ZnO under simulated sunlight.
• The photocatalytic oxidation process by ZnO nanoparticles can be used as a proper and environmentally friendly method for removing of low concentrations of VOCs from polluted air under sunlight.

## 1 Data

**Fig. 1:**
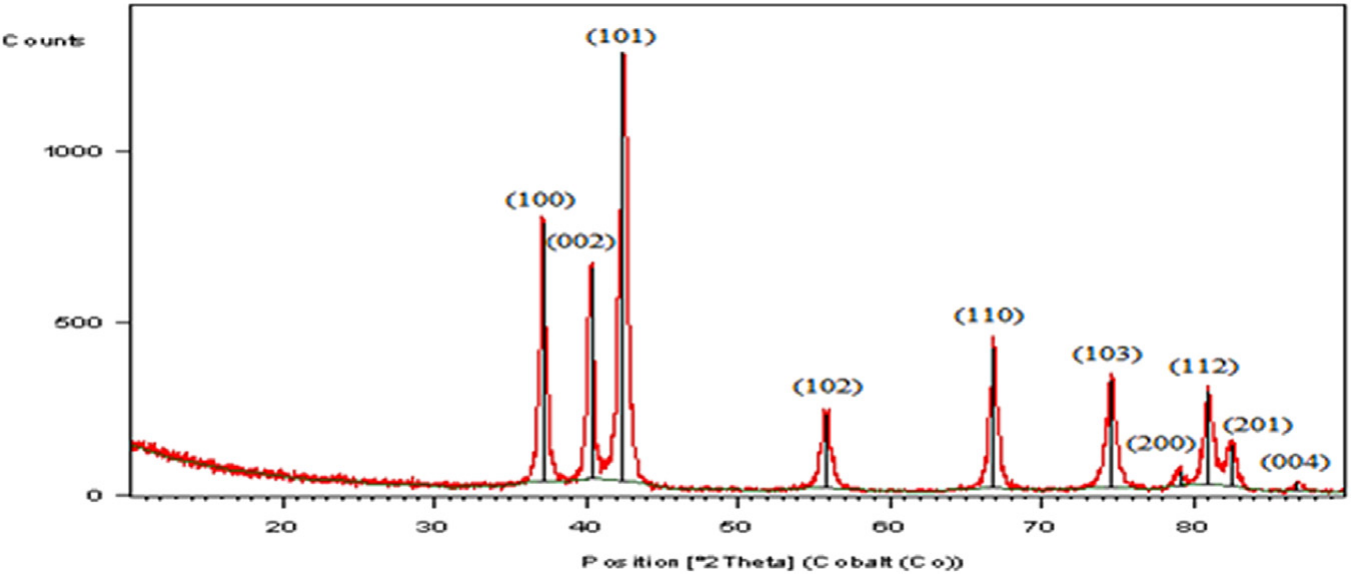
XRD pattern of ZnO.

**Fig. 2:**
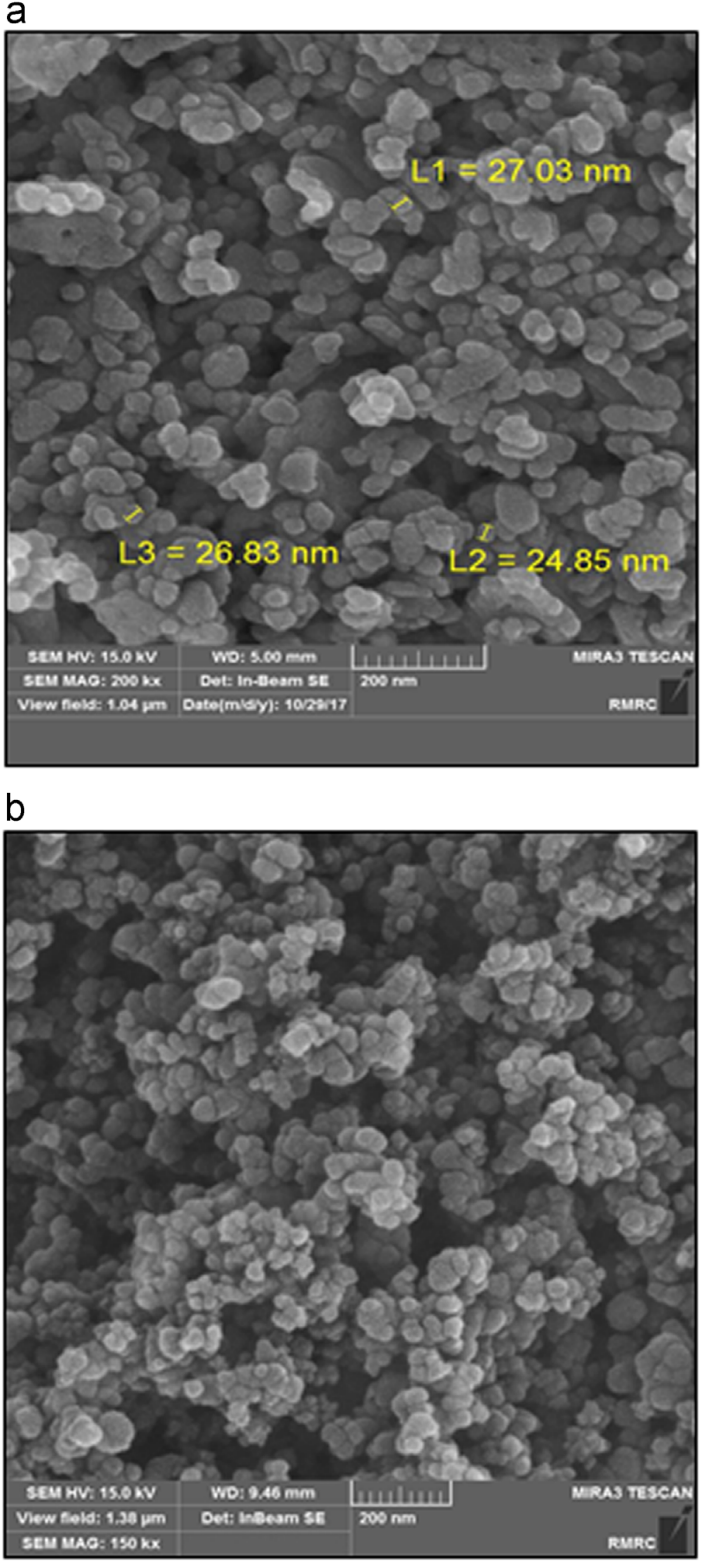
(a) SEM image of ZnO nanoparticles, (b) FE-SEM image of ZnO coated on glass plate.

The XRD pattern of ZnO powders is shown in Fig. 1. Nine diffraction peaks of ZnO nanoparticles are found at 37.132–86.896° corresponding to ZnO crystal phase (JCPDS 5-0664). The structural properties of the surface and mean size of the nanoparticles were determined by scanning electron microscopy (SEM) (Fig. 2a). Furthermore, after coating the nanoparticles on the glass plates, the structural properties of the immobilized nanoparticles were characterized using field emission scanning electron microscopy (FE-SEM) (MIRA3 TESCAN) (Fig. 2b).

**Fig. 3:**
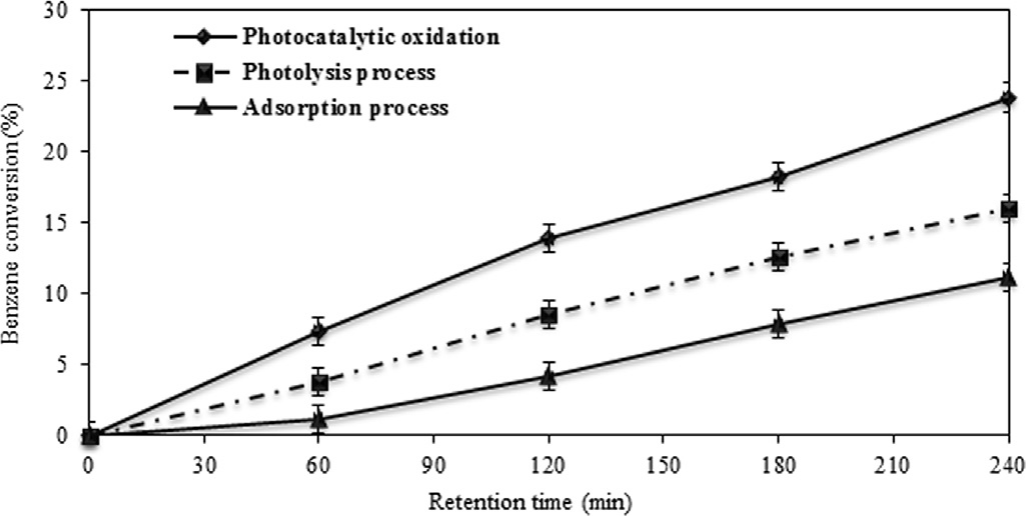
Benzene conversion by photocatalytic oxidation, photolysis, and adsorption process. Initial toluene concentration 50 ppm, at 25 °C, relative humidity 10%, and ZnO suspension dosage 10 g/L.

**Fig. 4:**
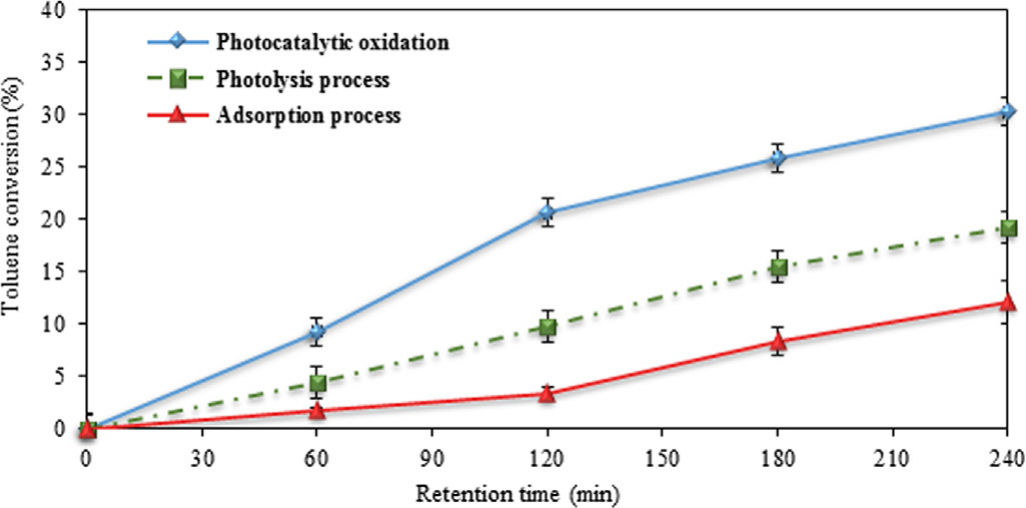
Toluene conversion by photocatalytic oxidation, photolysis, and adsorption process. Initial toluene concentration 50 ppm, at 25 °C, relative humidity 10%, and ZnO suspension dosage 10 g/L.

**Fig. 5:**
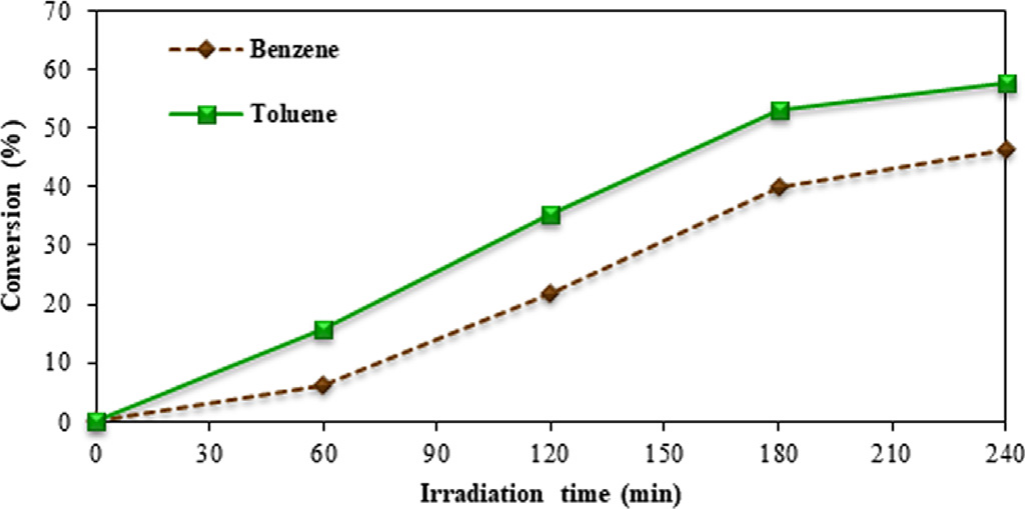
Photocatalytic oxidation of benzene and toluene mixture. Initial pollutants concentration 50 ppm, 45 °C, relative humidity 40%, ZnO suspension dosage 12 g/L.

Gas chromatography conditions for measuring pollutants are described in Table 1. Benzene and toluene conversion by photocatalytic oxidation, photolysis, and adsorption process are shown in Figs. 3 and 4. Photocatalytic oxidation of binary mixture is indicated in Fig. 5.

**Table 1:** Gas chromatography conditions for measuring pollutants.

Injection temperature (°C)	250
Detector temperature (°C)	250
Initial temperature of column (°C)	50
Increase the temperature (°C/min)	15
Second temperature of column (°C)	150

## 2 Experimental design, materials and methods

### 2.1 Materials

The chemical compounds used included benzene (purity 99.55, Code: B0143), which was purchased from Samchun Co. (South Korea), and toluene (purity 99.9%, CAS no.: 108-88-3), made by Merck Co. (Germany). They were used to synthesize the standard solution and prepare artificial polluted air entering the reactor. In addition, to extract benzene and toluene compounds from charcoal tube, carbon disulfide (CS_2_) prepared by Darkozist industrial and mineral research center was used. In order to prepare zinc oxide slurry, double distilled water was employed. To wash the glass surfaces, NaOH (Merck Co., CAS no.: 1310-73-2) was used. The ZnO nanoparticles was prepared from US Research Nanomaterials.

### 2.2 Coating of ZnO on glass plates

To immobilize ZnO powders on the glass plates, heat attachment method was used [1], [2]. In the first step, ZnO suspension was prepared by adding ZnO powders to distilled water across different concentrations regarding the values considered in the research. In the second step, the prepared suspension was stirred for 30 min on a magnetic stirrer. Then, it was sonicated in an ultrasonic bath (Elmasonic S 80/H, frequency 37 kHz) to completely separate ZnO nanoparticles and obtain a more uniform solution.

The tempered glass plates were used across three different sizes (22 * 25, 12 * 25, 17 * 25 cm^2^). Before immobilization, the mentioned plates were washed in a NaOH (0.01 M) solution to elevate OH groups and for better contact between ZnO and the plates. They were then washed by distilled water. Afterward, the prepared ZnO suspension was poured on the glass plates. The coated plates were first dried at 120 °C for 1 h in an oven and then calcinated in an electronic furnace (Fanazma Gostar Co., Iran, frequency 50 Hz) for 3 h at 450 °C. The immobilization process was performed three times to increase the loaded ZnO on the plates.

### 2.3 Photocatalytic experiments

The photocatalytic oxidation of benzene and toluene were carried out in a rectangular reactor made of Plexiglass with a volume of 15.6 L (30 cm * 26 cm * 20 cm). To uniform the airflow inside the reactor, a fan was installed in its upper part. The irradiation source was a 150 w metal halide lamp (345–800 nm), which was placed in the upper part of the reactor and inside a double-walled quartz jacket. The intensity of ultraviolet radiation in all experiments was 139 μw/cm^2^ measured by a UV-meter, while the intensity of illumination was measured by a luxmeter as 36,200 lx. To control the temperature of the experiments, the reactor was placed inside an ice bath. Furthermore, to prevent the effect of other lights in the laboratory environment and focus the whole lamp radiation into the reactor, the reactor was covered with aluminum sheet.

To supply the air entering the reactor, an air pump (Resun AC-9906) was used. The pump׳s airflow was divided into two parts, part of it entered the impinger containing the pollutant liquid to supply contaminated air [3]. The rest of the flow produced by the air pump was entered into the bubbler containing water, humidity was adjusted by hygrometer that installed in the reactor.

The vapors produced from the impinger and the humidifier entered the mixing chamber (Mariotte׳s bottle with a volume of 15 L) equipped with a stirrer to uniform the inlet flow to the reactor. The flow resulting from the mixing chamber entered the reactor after re-controlling the flow rate by the rotameter. All of the experiments were performed under laboratory hood to better control the environmental conditions of the reaction and prevent contamination of the laboratory environment.

In all experiments, after the time which had been obtained experimentally considering the flow rate entering the reactor and the volume of reactor, the inlet and outlet of the reactor were closed, and after reaching equilibrium of the flow inside the reactor, the reactor was sampled [4]. Sampling and analysis of the flow containing the pollutant were performed according to instruction 1501 NIOSH.

To evaluate the photolysis of the pollutant and adsorption of pollutant on glass plates, a set of controlling experiments were conducted, (1) in the presence of light and absence of catalytic plates and (2) in the absence of light and presence of catalytic plates. Furthermore, photocatalytic removal of pollutants was evaluated under similar conditions with control experiments conditions [5], [6], [7].

## References

[1] Behnajady M.A., Moghaddam S.G., Modirshahla N., Shokri M. (2009). Investigation of the effect of heat attachment method parameters at photocatalytic activity of immobilized ZnO nanoparticles on glass plate. Desalination.

[2] S. Hosseini, M. Borghei, M. Vossoughi, N. Taghavinia, Photocatalytic degradation of phenol in aqueous phase with TiO_2_ immobilized on three different supports with a simple method, in: Proceedings of the 3rd IASME/WSEAS International Conference on Energy & Environment, 2008, pp. 46–50.

[3] Jafari A.J., Kalantary R.R., Esrafili A., Arfaeinia H. (2018). Synthesis of silica-functionalized graphene oxide/ZnO coated on fiberglass and its application in photocatalytic removal of gaseous benzene. Process Saf. Environ. Prot..

[4] Jafari A.J., Kermani M., Kalantary R.R., Arfaeinia H. (2017). Photocatalytic abatement of o-xylene using adsorption enhanced ZnO/GAC catalyst in a continuous flow reactor: catalytic potential. Glob. NEST J..

[5] Rismanchian M., Akbari J., Keshavarzi R. (2014). Photocatalytic removal of gaseous toluene by titanium dioxide coated on nickel foam: influence of relative humidity and toluene concentration. Int. J. Environ. Health Eng..

[6] Soltani R. Darvishi Cheshmeh, Rezaee A., Safari M., Khataee A., Karimi B. (2015). Photocatalytic degradation of formaldehyde in aqueous solution using ZnO nanoparticles immobilized on glass plates. Desalin. Water Treat..

[7] Leili M., Farjadfard S., Sorial G.A., Ramavandi B. (2017). Simultaneous biofiltration of BTEX and Hg° from a petrochemical waste stream. J. Environ. Manag..

